# Development of efficient betaine-based natural deep eutectic solvents for metal leaching from waste printed circuit boards

**DOI:** 10.1007/s11356-026-38292-9

**Published:** 2026-10-20

**Authors:** Emmanuel Anuoluwapo Oke, Johannes Hermanus Potgieter

**Affiliations:** Sustainable and Innovative Minerals and Metals Extraction Technology (SIMMET) Research Group, School of Chemical and Metallurgical Engineering, University of the Witwatersrand Johannesburg, Private Bag X3 PO Wits 2050, Johannesburg, South Africa

**Keywords:** E-waste, Sustainable metal recovery, Natural deep eutectic solvents, Lixiviants, Copper, Dissolution kinetics

## Abstract

The rapid growth of electronic waste has intensified the need for sustainable approaches for metal recovery from waste printed circuit boards (WPCBs). In this study, betaine-based natural deep eutectic solvents (NADES) were developed as lixiviants for metal extraction from WPCBs. Betaine (BET) was used as the hydrogen bond acceptor, while ethane-1,2-diol (ETD), propane-1,2-diol (PPD), and butane-1,2-diol (BTD) served as hydrogen bond donors (HBDs) to investigate the influence of HBD structure on the physicochemical properties and leaching performance of the resulting NADES. Fourier transform infrared spectroscopy (FTIR) indicated intermolecular interactions between BET and the diol-based HBDs, while thermogravimetric analysis/derivative thermogravimetry (TGA/DTG) showed pronounced mass-loss-rate maxima above 240 °C for all three NADES. Rheological measurements revealed Newtonian flow behaviour, with viscosity increasing with increasing diol chain length; BET:ETD exhibited the lowest viscosity (50.1 mPa·s) at 20 °C. Under the experimental conditions of 75 °C, 6 h, 1.0 M H_2_O_2_, 500 rpm, and a solid-to-liquid ratio of 1:20, BET:ETD achieved leaching efficiencies of 99.3% for Cu, 99.5% for Pb, 97.8% for Fe, 95.4% for Al, 90.5% for Sn, and 73.9% for Ti. Kinetic analysis based on the shrinking core model and activation energy evaluation indicated a mixed-control mechanism involving interfacial reaction and diffusion processes. SEM, XRD, and FTIR analyses further indicated substantial dissolution of metallic phases while the polymeric and glass-fibre matrix was largely preserved. Overall, the results demonstrate that betaine-based NADES, most especially BET:ETD, offer tunable physicochemical properties and effective metal-leaching performance for WPCB recycling.

**Supplementary Information:**

The online version contains supplementary material available at https://doi.org/10.1007/s11356-026-38292-9.

## Introduction

The global surge in electronic waste (e-waste) is a direct consequence of the rapid growth within the integrated circuit industry and increasing demand for new electronic gadgets by consumers (Li et al. 2025; Peng et al. 2026). Market trends characterised by swift replacement cycles and relentless technical progress have drastically reduced the longevity of electronic hardware, placing immense strain on international waste management frameworks. A significant proportion of this waste contains hazardous components, most notably toxic heavy metals like lead, cadmium, and mercury, which are typically found in high concentrations within waste printed circuit boards (WPCBs). Due to their environmental persistence and high potential for bioaccumulation, these metals present a grave risk to both human health and natural ecosystems (Ashiq et al. 2019). Inadequate handling or disposal of WPCBs allows these heavy metals to permeate the environment, polluting groundwater and soil, damaging food chains, and causing lasting ecological harm.

According to the Global E-waste Monitor 2024, the world produced approximately 62 million metric tonnes of e-waste in 2022 (Baldé et al. 2024). Projections from the United Nations University (UNU) indicate that this volume could reach roughly 74.7 million metric tonnes by 2030 (Forti et al. 2020). This rate of expansion outpaces many other types of traditional waste. WPCBs are a pivotal element of the e-waste stream, accounting for an estimated 3.0–5.0% of the total weight (Oke and Potgieter 2024). Their physical makeup generally consists of 40.0% metals, 30.0% polymers, and 30.0% refractory materials (Annamalai and Gurumurthy 2021). These components house significant amounts of useful metals, including Cu, Al, Sn, Fe, Zn, Ni and Pb; notably, the concentration of precious metals in these boards is frequently nearly ten times higher than that found in natural ores (Aral and Beşe 2024). Furthermore, the inclusion of minor rare earth elements reinforces the status of WPCBs as a vital "urban mining" asset (Rao et al. 2020). The metallic portion of typical WPCB consists of a base metal fraction including about 20.0% Cu, 8.0% Fe, 4.0% Sn, 2.0% Pb, 2.0% Ni, and 1.0% Zn, as well as a precious metal component including 0.2% Ag, 0.1% Au, and 0.005% Pd (Singh et al. 2026). Despite the high value of these materials, the effective management of e-waste remains a significant global challenge. Even in nations with advanced waste management facilities, the complex nature of electronic scrap complicates processing efforts (Patil and Ramakrishna 2020). Consequently, there is an urgent need to recycle e-waste to recover these critical metals, thereby diverting hazardous materials from landfills and contributing to resource recovery and the circular economy. This is expected to help in the preservation of natural assets such as mineral ores, timber, and water, alongside economic advantages gained through the reuse of resources (Peng et al. 2026). Moreover, efficient recycling reduces the pollution caused by substandard disposal methods and stimulates employment within the recovery sector (Yong et al. 2019). Altogether, these factors are essential for advancing the principles of a circular economy within the electronics industry.

Conventional physical separation methods for separating metals from WPCB, including electrostatic separation, froth flotation, and the vibrating gas–solid fluidised bed technique, have been widely applied to achieve efficient separation of metallic and non-metallic fractions (Moganti and Dutta 2026). Furthermore, several metallurgical routes, namely pyrometallurgy, hydrometallurgy and bio-metallurgy, are commonly employed for the effective recovery of valuable metals (Dutta et al. 2023). Pyrometallurgical processing relies on differences in melting and boiling temperatures at elevated conditions to separate metallic from non-metallic constituents (Suffia and Dutta 2024; Zou et al. 2024). Despite its effectiveness in metal recovery, pyrometallurgy presents several limitations when applied to WPCBs. These include high energy consumption, the release of hazardous gases and the potential loss of valuable metals arising from inefficient extraction conditions. Furthermore, biometallurgical approaches utilise microorganisms to mobilise metals from solid printed circuit boards by converting them into soluble species in aqueous media through the production of lixiviants (Zhang et al. 2025). This method is attractive due to its environmentally benign nature, operation under mild conditions, cost-effectiveness, and low generation of secondary waste (Ji et al. 2022). However, its practical application remains limited by slow kinetics, as well as insufficient scientific understanding, most especially regarding optimal operating conditions and the integration of acidic and alkaline bio-based systems for large-scale implementation (Magoda and Mekuto 2022).

In addition, traditional hydrometallurgical treatment of WPCBs involves a sequence of pre-treatment, selective leaching, solution purification and subsequent metal recovery (Moganti and Dutta 2026; Zhang et al. 2025). Under relatively mild conditions, inorganic acids can effectively dissolve a broad range of metals, although the process is often limited by slow kinetics, leading to extended leaching durations before metal-rich solutions are obtained (Hao et al. 2020). To enhance extraction efficiency, oxidising agents such as I_2_, H_2_O_2_, K_2_Cr_2_O_7_, KMnO_4_, Cu^2+^ and Fe^3+^ are frequently employed. A study by Jadhao et al. reported up to 90.0% Ni and complete Cu (100.0%) recovery using ammonium–ammonia solutions containing 0.4 M H_2_O_2_ (Jadhao et al. 2021). However, the dependence on volatile, highly acidic and toxic reagents, including cyanide and thiosulphate systems, presents serious environmental and safety concerns, thereby constraining industrial implementation and motivating the development of more sustainable alternatives (Moganti and Dutta 2026).

Deep eutectic solvents (DESs) have emerged as promising alternatives to conventional solvents due to their ability to dissolve metal oxides and salts, making them highly suitable for leaching applications (Moganti and Dutta 2026; Suffia and Dutta 2024; Yuan et al. 2022). Their growing adoption is driven by increasing concerns over the toxicity and volatility of traditional organic solvents, which pose significant environmental and health risks (Dai et al. 2013; Tran et al. 2019). Most importantly, natural deep eutectic solvents (NADES), synthesised from bio-derived constituents, offer additional advantages such as low toxicity, biodegradability, low cost, sustainability and straightforward preparation (Dai et al. 2015). They are prepared by combining two components, a hydrogen bond acceptor (HBA) and a hydrogen bond donor (HBD), creating a mixture that possesses a lower melting point than its individual constituents (Ijardar et al. 2022). In the context of WPCB treatment, the reported application of DESs has predominantly involved choline chloride (ChCl)-based systems. ChCl-based DESs containing ethane-1,2-diol, oxalic acid and glycolic acid have been investigated for the recovery of Zn, Pb and Cu from WPCBs, while formic acid–ChCl, urea–ChCl and oxalic acid-based systems have also been evaluated for metal recovery from WPCBs (Mishra et al. 2024; Zhao et al. 2023). These studies demonstrate the potential of DESs as alternative lixiviants for WPCB treatment, but the reported systems have largely centred on ChCl as the HBA.

Furthermore, betaine has also been investigated as an alternative HBA for the formation NADES. Several betaine-based NADES, including betaine–1,2-propanediol systems, have been characterised in terms of their physicochemical and thermal properties, while the influence of composition on the properties of betaine–1,2-propanediol systems has also been investigated (Chen et al. 2022; Fuad and Nadzir 2022). Betaine-based NADES have additionally demonstrated potential for metal extraction from other secondary resources, including the leaching of Li, Co, Ni and Mn from spent lithium-ion battery cathode materials using a betaine hydrochloride–citric acid NADES (Luo et al. 2023). However, based on the available literature, the application of betaine-based NADES specifically to the leaching of metals from WPCBs remains largely unexplored. Moreover, the influence of systematic structural variation of HBDs on the physicochemical properties and metal-leaching performance of betaine-based NADES has not been established for WPCB feedstocks. This represents an important knowledge gap because changes in HBD molecular structure can modify viscosity, intermolecular interactions and mass-transfer behaviour, which may consequently influence metal dissolution from the heterogeneous WPCB matrix.

To address this gap, the present study investigates betaine (BET) as the HBA in combination with three structurally related diol HBDs, namely ethane-1,2-diol (ETD), propane-1,2-diol (PPD), and butane-1,2-diol (BTD), for the leaching of Cu, Al, Fe, Sn, Pb and Ti from WPCBs. The progressive increase in HBD alkyl-chain length provides a systematic basis for evaluating the relationship between HBD structure, NADES physicochemical properties and metal-leaching performance. The study therefore seeks to establish structure–property–performance relationships that can inform the development of tunable and sustainable solvent systems for metal recovery from complex WPCB feedstocks.

## Materials and methods

### Materials

BET (C_5_H_11_NO_2_·H_2_O) was sourced from Sigma-Aldrich with a purity of ≥ 99.0%. Also, ETD (C_2_H_6_O_2_) with a purity of > 99.9% and PPD (C_3_H_8_O_2_) with a purity of > 99.5% were obtained from Associated Chemical Enterprises Pty. Additionally, BTD (C_4_H_10_O_2_) with a purity of ≥ 98.0% was purchased from Sigma-Aldrich. Hydrogen peroxide (H_2_O_2_) with a concentration of 50.0% was acquired from Associated Chemical Enterprises Pty. Furthermore, iodine (I_2_) with a purity of ≥ 99.8%, potassium permanganate (KMnO_4_) with a purity of ≥ 99.0%, and ethanol (C_2_H_6_O) with a purity of ≥ 99.5% were all sourced from Sigma-Aldrich. Sodium hydroxide (NaOH) with a purity of 98.0% was acquired from Loba Chemie. All the chemicals utilised in this study were used as received without further purification. Deionised water was employed for all preparatory procedures and the cleaning of laboratory apparatus.

### Sourcing of WPCBs

HP desktop computer units were obtained from the University of the Witwatersrand, School of Chemical and Metallurgical Engineering, for experimental purposes. The WPCBs were manually dismantled from the discarded computer casings. Bulky and non-essential attachments, such as connectors and stainless-steel heat sinks, were deliberately removed to avoid complications during size reduction. Thereafter, the recovered boards were cut into smaller pieces using a saw to facilitate further processing.

### Pre-treatment and size reduction of WPCBs

For effective metal recovery, the removal of the protective solder mask is essential. This green epoxy-based coating serves as a resistant barrier against thermal and chemical attack, thereby limiting the access of lixiviants to the embedded metallic phases. To overcome this, the sectioned WPCBs were immersed in a 5.0 M NaOH solution for 24 h to chemically strip off the epoxy layer, following established hydrometallurgical procedures (Nagarajan and Panchatcharam 2023). Although NaOH is inherently caustic, its controlled use at this concentration remains a conventional and efficient approach for exposing metallic surfaces without undermining the overall sustainability of the process. Earlier studies have demonstrated that 5.0 M NaOH is adequate for complete resin removal, rendering higher concentrations such as 10.0 M superfluous (Nagarajan and Panchatcharam 2023; Oke and Potgieter 2025b). After treatment, the WPCBs were thoroughly washed with deionised water to remove residual alkalinity and then air-dried. The dried WPCB material was subsequently subjected to mechanical comminution and sieved to obtain particles smaller than 300.0 µm. This reduction in particle size is vital, as it enhances the liberation of metals from the complex WPCB matrix and increases the available surface area for efficient leaching.

### Characterisation of WPCBs

A comprehensive characterisation of the ground WPCB sample was performed using a suite of complementary analytical techniques. The bulk elemental composition was determined via X-ray fluorescence (XRF) spectroscopy using a Rigaku NEX DE instrument. To ensure analytical consistency, the ground sample was homogenised, mixed with a suitable binder, and pressed into pellets prior to analysis. The resulting primary elemental profile obtained is detailed in Table 1. The surface features and microstructural evolution of the WPCBs, as well as the corresponding leach residues, were examined using a Zeiss Sigma field emission scanning electron microscope (FE-SEM). Imaging was performed in backscattered electron mode at an accelerating voltage of 20 kV with a 60 mm aperture, enabling clear contrast between phases of varying atomic number. In addition, phase identification and mineralogical evaluation were carried out using X-ray diffraction (XRD) analysis. Measurements were performed on a Bruker D8 ADVANCE diffractometer with Cu-Kα radiation (λ = 1.54060 Å). The diffraction patterns were interpreted using the PDF-2 RELEASE 2016 RDB database, allowing for the identification of constituent phases and monitoring of structural transformations throughout the extraction process.

**Table 1:** Bulk elemental composition of raw WPCB samples determined by XRF analysis

Cu (mass %)	Al (mass %)	Fe (mass %)	Sn (mass %)	Pb (mass %)	Ti (mass %)
36.10	17.30	7.24	8.61	1.21	1.26

### Synthesis of NADES

**Fig. 1:**
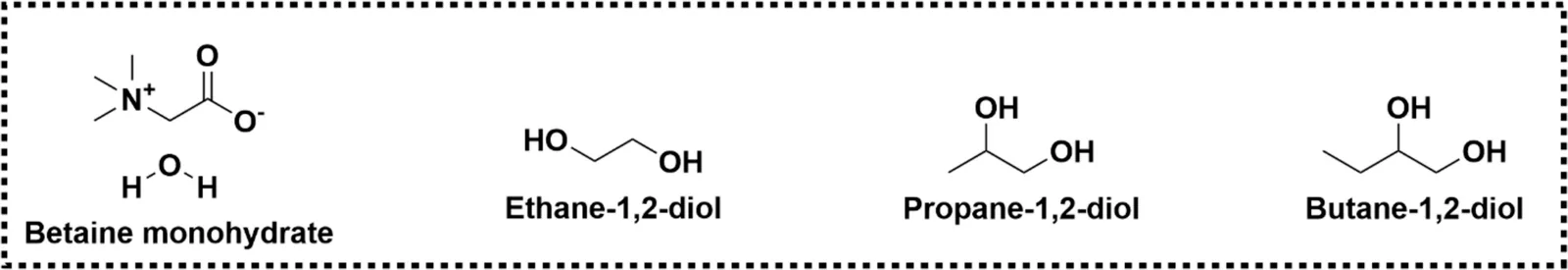
Chemical structures of NADES components used in this study

The NADES used in this study (BET:ETD, BET:PPD, and BET:BTD) were synthesised by mixing BET with the respective 1,2-diols (ETD, PPD and BTD) and deionised water in a 1:3:1 molar ratio. The BET:HBD molar ratio of 1:3:1 was selected following the formulation adopted for betaine-based diol systems in previous work (Koh and Khoiroh 2024; Yue et al. 2021). The ternary mixtures were subjected to constant agitation at 500 rpm on a digital hotplate maintained at 70 °C. Heating was maintained until the components transitioned into a single-phase, transparent liquid, signifying the completion of the eutectic formation. To ensure the exclusion of atmospheric moisture and maintain chemical stability, the prepared NADES were stored in a desiccator for 5 days before being transferred into sealed glass vials for subsequent use. No additional purification was required, and the chemical structures of the constituents of the NADES are illustrated in Fig. 1.

### Characterisation of the NADES

The chemical structure and intermolecular interactions within the synthesised NADES were probed using Fourier transform infrared spectroscopy (FTIR) on a Perkin Elmer Spectrum Two instrument. Spectra were recorded over the range of 4000–400 cm⁻^1^ at a resolution of 4 cm⁻^1^, with four successive scans collected for each sample. This analysis facilitated the identification of functional groups and provided evidence of hydrogen-bonding interactions responsible for eutectic formation, while also confirming the absence of unintended chemical alterations.

In addition, thermal behaviour of the prepared NADES was investigated by thermogravimetric analysis (TGA) using a Mettler Toledo TGA 2 instrument. Approximately 10 mg of each sample was placed in alumina crucibles and subjected to a controlled heating programme from 25 °C to 500 °C. The analysis was carried out under a nitrogen atmosphere at a flow rate of 30 mL min⁻^1^, with a constant heating rate of 10 °C min⁻^1^. This assessment enabled the determination of thermal limits, ensuring that the solvents maintain structural stability under operational conditions.

Furthermore, the rheological properties of the investigated NADES were examined using an Anton Paar MCR 102 modular rheometer equipped with a CP 40 cone-and-plate measuring system. The geometry consisted of a 1° cone angle with a truncation gap of 0.1 mm, while temperature control was maintained via a Peltier system. Measurements were conducted at 20 °C at a shear rate interval of 1.0–100.0 s⁻^1^ under steady-state conditions. In addition, the measurements were performed using the as-prepared NADES formulations containing the water incorporated during NADES formation at a BET:HBD:water molar ratio of 1:3:1, without subsequent drying or additional water addition. This evaluation provided insight into the flow behaviour of the prepared NADES and yielded viscosity data relevant to their handling and mass transport behaviour.

### Leaching procedures

The leaching experiments were conducted by employing a 100 mL two-necked round-bottom flask with a specific mass of ground WPCB to maintain a constant solid-to-liquid (S:L) ratio of 1:20 (w/w). This is consistent with our previously established method which provided good leaching efficiency (Oke et al. 2025). The reactor was equipped with a water-cooled reflux condenser and submerged in a thermostatically regulated water bath to ensure temperature stability. Uniform mixing of the suspension was achieved through continuous magnetic stirring. To evaluate the influence of thermodynamic and kinetic variables on metal recovery, the leaching temperature was varied across a discrete range (25, 35, 45, 55, 65, 75, and 85 °C), while the residence time was adjusted between 1 and 6 h. Following each leaching interval, the mixture was subjected to vacuum filtration using a Büchner funnel fitted with Whatman No. 42 filter paper. The resulting solid residue was thoroughly washed with a 40.0% ethanol solution to recover any entrained NADES and remove superficial impurities. The collected leachates were subsequently prepared for elemental analysis to determine the concentrations of dissolved metals using inductively coupled plasma mass spectrometer (ICP-MS 7700, Agilent, USA). To ensure the reliability of the analytical data, all experiments were performed in duplicate, and the mean values were reported. The leaching efficiency for each target metal was calculated according to Eq.(1).

1 $$Leaching\;efficiency\left(\%\right)=\frac{P\times Q}{R\times S}\times100$$

where *P* represents the concentration of the target metal in the leachate, *Q* denotes the leachate volume, *R* symbolises the amount of the target metal in the original sample, and *S* designates the sample weight.

## Results and discussion

### FTIR characterisation of the NADES

**Fig. 2:**
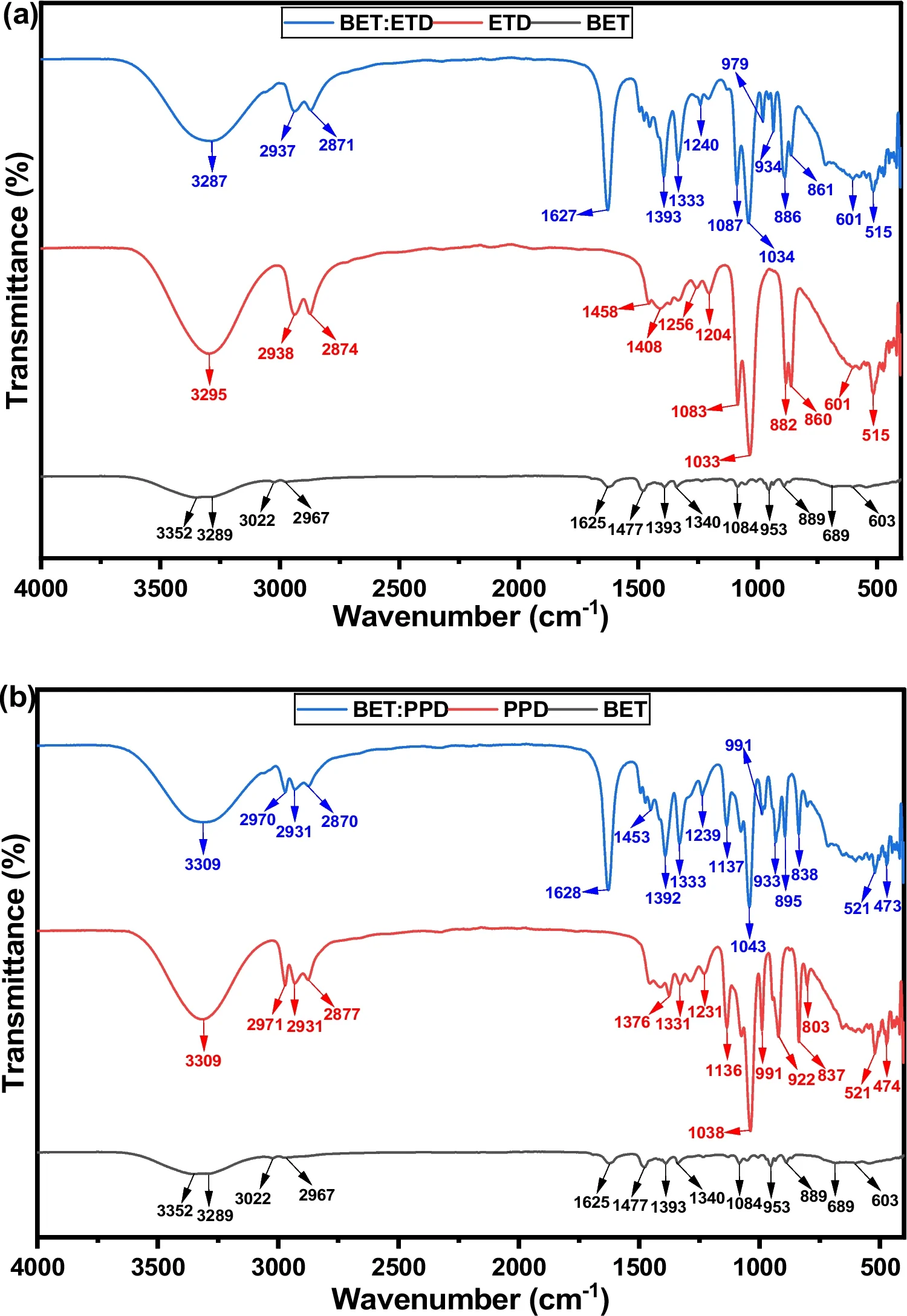

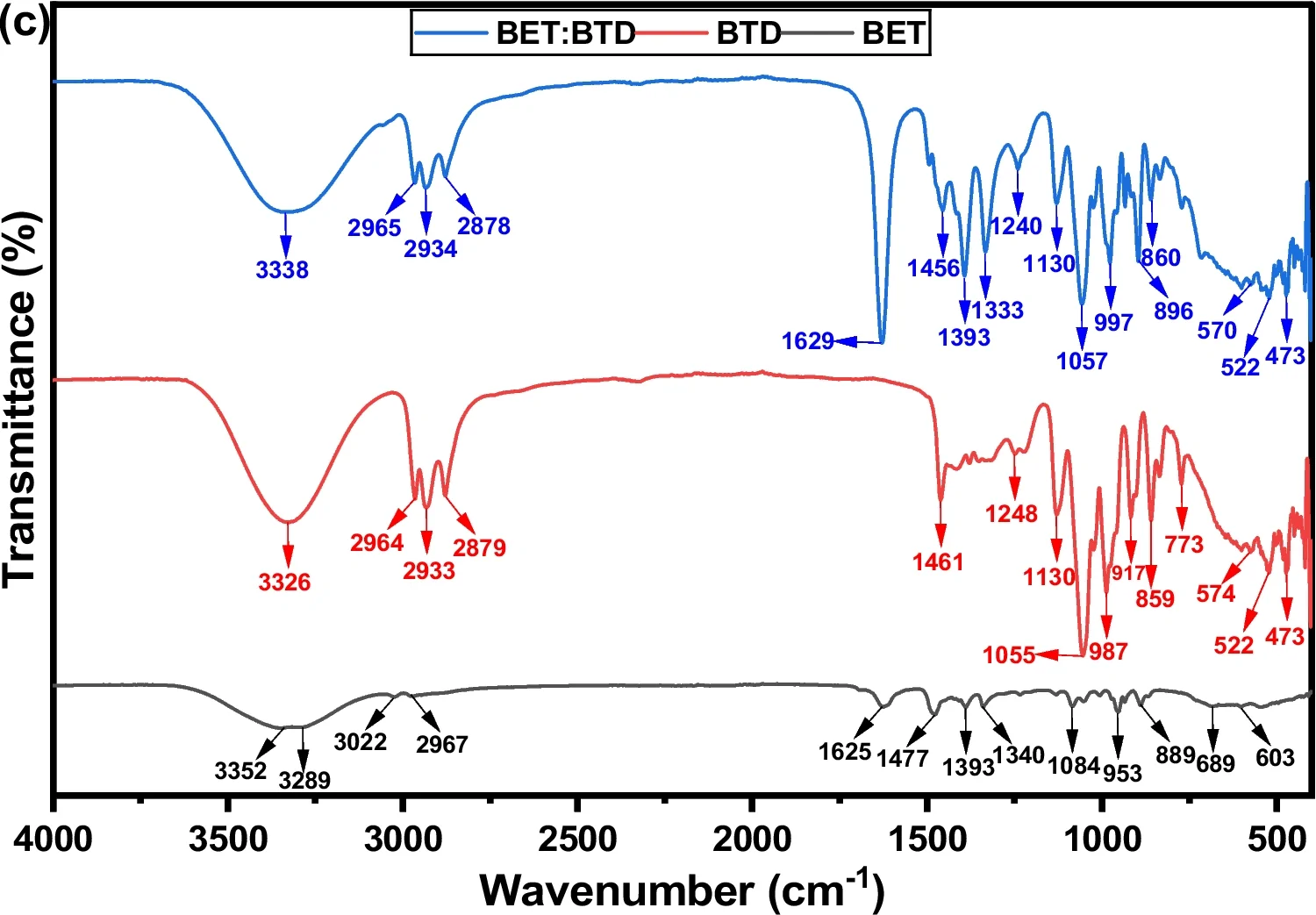
FTIR spectra of **a** BET, ETD, and BET:ETD NADES; **b** BET, PPD, and BET:PPD NADES; and **c** BET, BTD, and BET:BTD NADES

FTIR spectroscopy was employed to provide molecular-level evidence of the successful formation of betaine-based NADES and to highlight the specific hydrogen-bonding sites between the HBA (BET) and HBDs (ETD, PPD, and BTD). The comparative infrared spectra for the pure components of the NADES and their resulting eutectic mixtures are illustrated in Fig. 2a–c. In general, –OH stretching vibrations are usually observed within the 3700–3100 cm⁻^1^ region and are highly sensitive to hydrogen-bonding interactions. Variations in band position and broadness are indicative of changes in hydrogen-bond strength and are widely used as primary evidence for DES formation (Basaiahgari et al. 2018; Ghaedi et al. 2017).

For pure BET, the most critical vibrations include the O–H stretching of the water of crystallisation at 3352 cm⁻^1^ and 3289 cm⁻^1^, alongside the characteristic vibrations of the zwitterionic structure. The aliphatic C–H stretching vibrations appear at 3022–2967 cm⁻^1^, corresponding to the methyl groups of the quaternary ammonium centre. The intense band at 1625 cm⁻^1^ is assigned to the asymmetric stretching of the carboxylate group (–COO⁻), while the corresponding symmetric stretching is identified near 1340 cm⁻^1^. The quaternary ammonium headgroup is represented by the C–N⁺ symmetric stretching vibration at 953 cm⁻^1^, and the structural integrity of the hydrocarbon backbone is confirmed by the –CH_2_ scissoring (bending) mode at 1477 cm⁻^1^. Upon the formation of the NADES, the –COO⁻ asymmetric peak at 1625 cm⁻^1^ undergoes distinct broadening and a perceptible shift, providing definitive evidence that the carboxylate oxygens act as primary hydrogen-bond acceptors for the hydroxyl protons of the diols. A detailed examination of the high-frequency region reveals that the pure diols exhibit intense, broad bands corresponding to self-associated O–H stretching vibrations. In the BET:ETD NADES (Fig. 2a), this band is centred at 3287 cm⁻^1^, representing a clear red shift from the pure ETD vibration of 3295 cm⁻^1^. Similarly, the O–H stretching vibrations for BET:PPD NADES (Fig. 2b) and BET:BTD (Fig. 2c) are recorded at 3309 cm⁻^1^ and 3338 cm⁻^1^, respectively, representing a clear blue shift from the pure BTD vibration of 3326 cm⁻^1^. These displacements, coupled with the enhanced breadth of the absorption envelopes, signify a transition from the homo-molecular H-bonds of the pure diols to a complex, inter-molecular H-bonding network with the BET molecules. Complementing these shifts, the asymmetric and symmetric C–H stretching vibrations appearing between 2937 cm⁻^1^ and 2871 cm⁻^1^ correspond to the sp^3^ hybridised carbons in both the BET alkyl chain and the diol backbones. Furthermore, the intense absorption bands located at 1034 cm⁻^1^ (BET:ETD), 1043 cm⁻^1^ (BET:PPD), and 1057 cm⁻^1^ (BET:BTD) are assigned to the C–O stretching of the alcohol groups. The preservation and sharpness of these peaks, alongside the consistent presence of the C–N⁺ marker at 953 cm⁻^1^, confirm that the constituents remain chemically intact and do not undergo degradation or side reactions during the thermal synthesis at 70 °C. Collectively, an intermolecular association was established between the –COO⁻ moiety of BET and O–H functionality of the HBDs. This interaction arises from hydrogen bond formation, whereby the highly electronegative O⁻ of the –COO⁻ group serves as the acceptor, interacting with the partially positive hydrogen atom of the O–H group (Li et al. 2020). The observed spectra of the prepared NADES show strong consistency with previously reported spectra for betaine-based NADES systems in the literature (Fuad and Nadzir 2022; Li et al. 2020; Mulia et al. 2019).

### Thermal stability of NADES

**Fig. 3:**
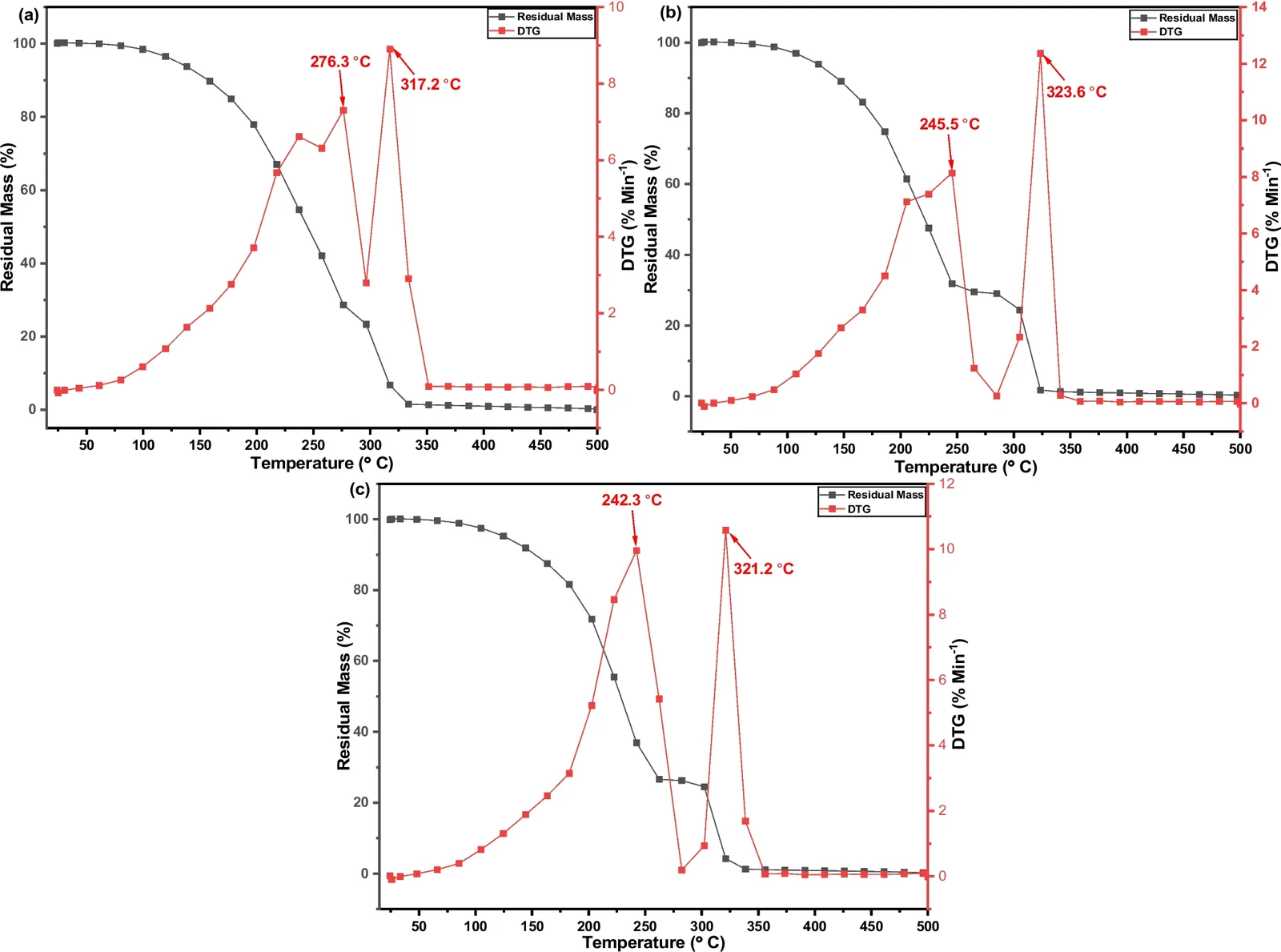
Thermal stability of the synthesised **a** BET:ETD, **b** BET:PPD, and **c** BET:BTD NADES

Figure 3a–c presents the TGA and corresponding derivative thermogravimetry (DTG) profiles of BET:ETD, BET:PPD and BET:BTD, respectively, over the temperature range of 25–500 °C. The TGA curves show the variation in residual mass with increasing temperature, while the DTG profiles indicate the temperatures at which the mass-loss-rate reaches pronounced maxima. Accordingly, the temperatures identified from the DTG profiles are discussed as maximum mass-loss-rate temperatures rather than onset decomposition temperatures. This distinction is important because a DTG maximum represents the temperature at which the rate of mass loss is greatest, whereas the onset temperature refers to the beginning of a detectable thermal mass-loss event (Delgado-Mellado et al. 2018; Fuad and Nadzir 2022). BET:ETD exhibits two pronounced DTG maxima at approximately 276.3 and 317.2 °C (Fig. 3a). The first maximum occurs after a broad region of progressive mass loss, followed by a second pronounced maximum at higher temperature. BET:PPD similarly exhibits progressive mass loss followed by pronounced DTG maxima at approximately 245.5 and 323.6 °C (Fig. 3b). A comparatively weak intermediate DTG feature occurs around 305.3 °C, but its substantially lower intensity relative to the two pronounced DTG maxima indicates that it is more appropriately considered an overlapping or shoulder feature rather than a distinct major thermal event. BET:BTD shows a comparable profile, with pronounced DTG maxima at approximately 242.3 and 321.2 °C and a weak intermediate feature around 302.3 °C (Fig. 3c). These temperatures correspond to the temperatures of maximum mass-loss-rate and do not indicate the temperatures at which mass loss initially begins. The TGA profiles demonstrate that substantial mass loss occurs before the first pronounced DTG maximum is reached. For example, the residual masses of BET:PPD and BET:BTD at their first pronounced DTG maxima are approximately 31.8% and 36.9%, respectively. Thus, the mass loss preceding the DTG maxima represents the cumulative effect of thermal mass loss occurring over the preceding temperature range, whereas the DTG maxima identify the temperatures at which the mass-loss processes reach their highest rates.

The thermal profiles further indicate that mass loss occurs over broad temperature ranges rather than through a single abrupt event. Such behaviour is consistent with previous studies of betaine-based and other eutectic systems, in which thermal degradation can involve contributions from the HBD, the interacting HBD–HBA structure and the HBA, with the corresponding processes occurring sequentially or in overlapping temperature ranges (Delgado-Mellado et al. 2018; Mero et al. 2023). Previous work on betaine- and choline-based DESs has identified multiple mass-loss events associated with evaporation or degradation of the HBD, degradation of the interacting HBD–HBA system and subsequent degradation of the HBA (Mero et al. 2023). The present results similarly indicate overlapping thermal processes; however, the individual DTG maxima cannot be assigned exclusively to specific components because the available TGA/DTG data do not directly identify the species responsible for each mass-loss event. In addition, a gradual mass loss is also evident below 150 °C in the present TGA profiles. This observation is relevant because deionised water was incorporated during NADES preparation at a BET:HBD:water molar ratio of 1:3:1. The low-temperature mass loss may therefore include the removal of water and other volatile species associated with the NADES matrix. However, the TGA data do not allow these contributions to be quantitatively separated. Consequently, the low-temperature region is interpreted as an overall mass-loss region rather than being attributed exclusively to water. Interestingly, water content and composition have been shown to influence the physicochemical and thermal behaviour of betaine-based NADES (Fuad and Nadzir 2022).

The thermal behaviour of the three NADES does not show a consistent dependence on the carbon chain length of the diol. For instance, the first pronounced DTG maximum occurs at 276.3 °C for BET:ETD, 245.5 °C for BET:PPD and 242.3 °C for BET:BTD, whereas the second maxima occur within the relatively narrow range of 317.2–323.6 °C. This variation indicates that the thermal behaviour of the NADES cannot be attributed to diol chain length alone, but rather reflects the combined effects of the composition and molecular structure of the constituent components. Overall, BET:ETD, BET:PPD and BET:BTD exhibit progressive and overlapping thermal mass-loss processes, with the two pronounced DTG maxima for each NADES occurring above approximately 240 °C. These results indicate that the NADES undergo their most pronounced mass-loss processes at temperatures substantially higher than those employed in the leaching experiments. However, measurable mass loss occurs at lower temperatures, and the DTG maxima cannot therefore be regarded as characteristic maximum mass-loss-rate temperatures rather than absolute limits of thermal stability.

### Flow behaviour of NADES

**Fig. 4:**
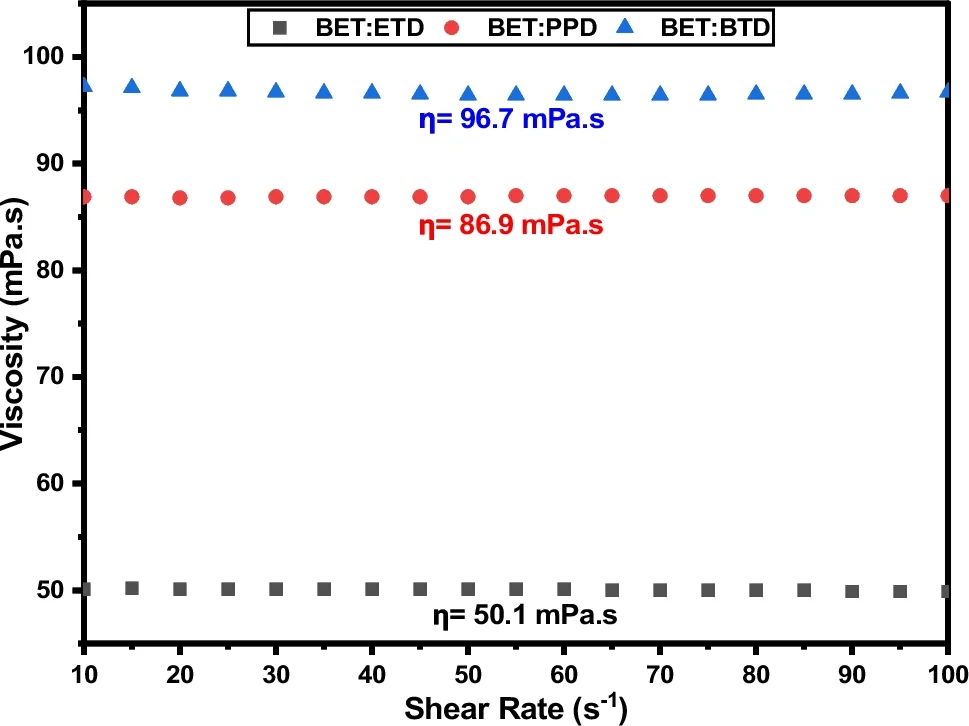
Viscosity (*η*) versus shear rate at a constant temperature of 20 °C and atmospheric pressure of 0.1 MPa for the synthesised NADES

Figure 4 illustrates the rheological behaviour of the synthesised betaine-based NADES (BET:ETD, BET:PPD, and BET:BTD) as a function of shear rate at 20 °C. The viscosity profiles show that all systems maintain nearly constant viscosity across the investigated shear rate range of 1 to 100 s⁻^1^, confirming that the prepared NADES exhibit Newtonian fluid behaviour, where viscosity is independent of the applied shear rate. The measured viscosities at 20 °C further reveal a clear dependence on the nature of HBD. Specifically, BET:BTD exhibits the highest viscosity of 96.7 mPa·s, followed by BET:PPD at 86.9 mPa·s, while BET:ETD shows the lowest viscosity among the three NADES. The increase in viscosity with increasing diol chain length is consistent with reported trends for betaine-based glycol DESs, in which viscosity depends strongly on the molecular structure of the HBD (Aravena et al. 2023). Similar observations have been widely reported, where the physicochemical properties of NADES are strongly governed by the structure and functionality of their constituent HBDs and HBAs (Aroso et al. 2017; Jangir et al. 2022; Oke and Potgieter 2025a). The viscosity of betaine-based glycol NADES is strongly dependent on both temperature and water content (Alcalde et al. 2024; Aravena et al. 2023). For instance, Aravena et al. reported a marked decrease in viscosity with increasing temperature for betaine-based systems containing ethylene glycol and other glycols (Aravena et al. 2023). Similar temperature-dependent behaviour has been reported for betaine:1,2-propanediol DESs (Chen et al. 2022). Water addition has also been shown to reduce the viscosity of betaine-based glycol DESs, although changes in water content can modify the intermolecular interactions and molecular organisation of the systems (Alcalde et al. 2024). In the present study, the rheological measurements were performed using the as-prepared NADES formulations containing the water incorporated during NADES formation at a BET:HBD:water molar ratio of 1:3:1, without subsequent drying or additional water addition. Additional water was not introduced because increasing the water content would alter the composition and intermolecular environment of the NADES (Alcalde et al. 2024); its effect on the leaching performance of the present system was not experimentally established. Thus, the viscosities measured in this study at 20 °C characterise the prepared NADES under the rheological measurement conditions and do not represent their viscosity at elevated operating temperatures.

Overall, the rheological results demonstrate that BET:ETD, BET:PPD and BET:BTD exhibit Newtonian flow behaviour with moderate viscosities at 20 °C. The observed viscosity differences among the three NADES reflect the influence of the diol structure, while the reported temperature dependence of betaine-based glycol DESs indicates that viscosity decreases substantially as temperature increases (Aravena et al. 2023; Chen et al. 2022). These characteristics indicate that the prepared NADES possess predictable flow behaviour under the conditions investigated and provide relevant information for assessing their handling and mass-transfer characteristics during subsequent application.

### Parametric investigation of leaching efficiencies

To achieve efficient recovery of the investigated metals WPCBs, a systematic leaching approach using betaine-based NADES was followed. A series of controlled experiments were conducted to evaluate the influence of key parameters on leaching performance. These include the alkyl chain length of the HBD component of the NADES, the nature of the oxidising agents (H_2_O_2_, KMnO_4_, and I_2_), leaching time, and operating temperature. A comprehensive discussion of the effects of these parameters on metal recovery efficiency is presented in this section.

#### Effect of HBD alkyl chain length in NADES

**Fig. 5:**
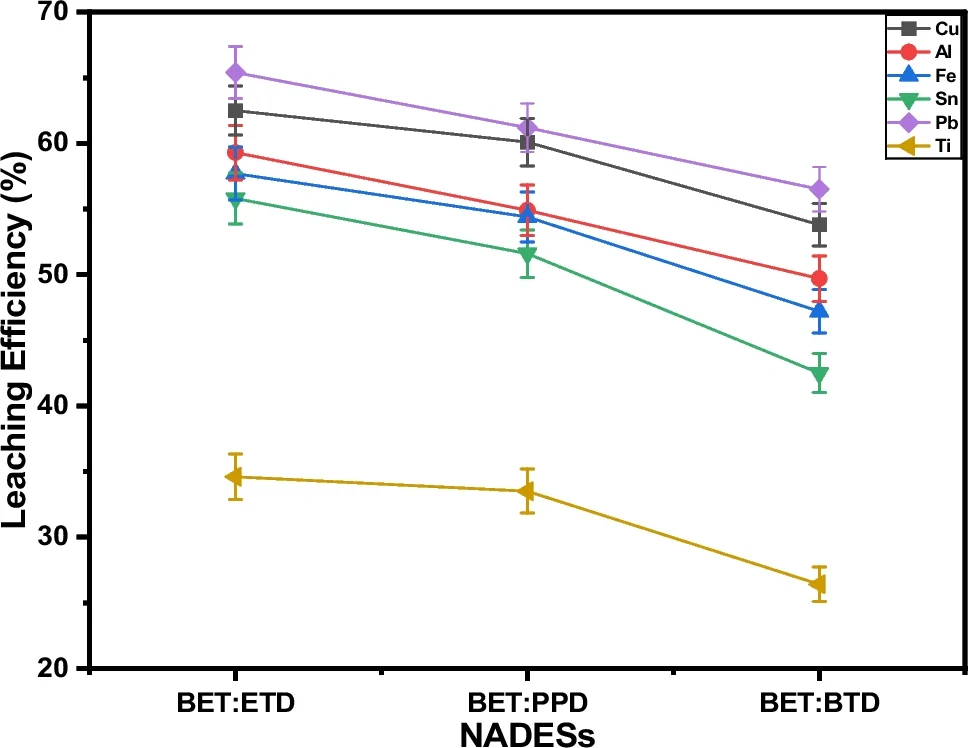
Effect of HBD alkyl chain length in NADES on the leaching of metals Influence of HBD on the leaching efficiency of metals [*T* = 55 °C; *t* = 4 h; stirring speed = 500 & S:L = 1:20]

The selection of the HBD plays a critical role in defining the physicochemical properties of NADES, which in turn govern their metal dissolution performance. It is well-established that the molecular structure of the HBD influences key parameters such as viscosity, hydrogen-bonding interactions, polarity, and metal coordination ability, all of which may collectively determine leaching efficiency (Ijardar et al. 2022; Moganti and Dutta 2026; Yuan et al. 2022). The effect of HBDs on leaching efficiency was investigated using BET-based NADES with increasing alkyl chain length, namely BET:ETD, BET:PPD, and BET:BTD. The leaching was performed for 4 h at 55 °C with a S:L ratio of 1:20 and a stirring speed of 500 rpm. The results obtained are presented in Fig. 5 and Table S1. For Cu, the highest recovery of 62.5% was obtained with BET:ETD, followed by 60.1% for BET:PPD and 53.8% for BET:BTD. A similar trend is observed for Al, where leaching decreases from 59.3% (BET:ETD) to 54.9% (BET:PPD) and further to 49.7% (BET:BTD). For Fe and Sn, the effect of increasing alkyl chain length is more pronounced. Fe recovery decreases from 57.7 to 54.4% and 47.2%, while Sn decreases from 58.8 to 51.6% and 42.5%, respectively. Pb follows the same trend, decreasing from 65.4% (BET:ETD) to 61.2% (BET:PPD) and 56.5% (BET:BTD). Ti shows the lowest recoveries overall and exhibits a decrease from 34.6 to 33.5% and 26.4%. Across all metals, BET:ETD consistently provides the highest extraction efficiency, while BET:BTD gives the lowest. This demonstrates that increasing the alkyl chain length of the HBD from ETD to BTD adversely affects metal dissolution under the studied conditions.

From a mechanistic standpoint, this behaviour reflects the interplay between solvent structure and transport properties. Short-chain diols such as ETD produce NADES with lower viscosity and higher polarity, which facilitate better wetting of the solid surface and more efficient diffusion of reactive species (Cotroneo-Figueroa et al. 2022; Sahin et al. 2026). In contrast, increasing the alkyl chain length to PPD and BTD introduces greater hydrophobic character and steric bulk, which can reduce solvent accessibility to active sites on the solid matrix (Yuan et al. 2022). In addition, as established in, viscosity increases with increasing HBD chain length. Higher viscosity in BET:BTD is expected to significantly limit mass transfer, reducing the rate at which solvated metal species can diffuse from the solid surface into the lixiviant. This results in lower effective reaction rates and reduced overall leaching efficiency. The strong and consistent decline observed for Fe, Sn, and Pb may suggest that these metals are more sensitive to diffusion limitations within the NADES matrix. Longer-chain HBDs may offer increased flexibility and potential coordination sites. However, it seems that this effect is insufficient to compensate for the adverse impact of reduced mass transport. Therefore, it can be inferred that the leaching process in this study is primarily controlled by physicochemical transport properties rather than enhanced coordination strength. Overall, the results demonstrate that shorter-chain HBDs are more favourable for metal extraction in BET-based NADES under the investigated conditions. BET:ETD provides the most efficient system due to its lower viscosity, higher polarity, and improved mass transfer characteristics, making it the most suitable solvent for subsequent studies.

#### Effect of the type of oxidants

**Fig. 6:**
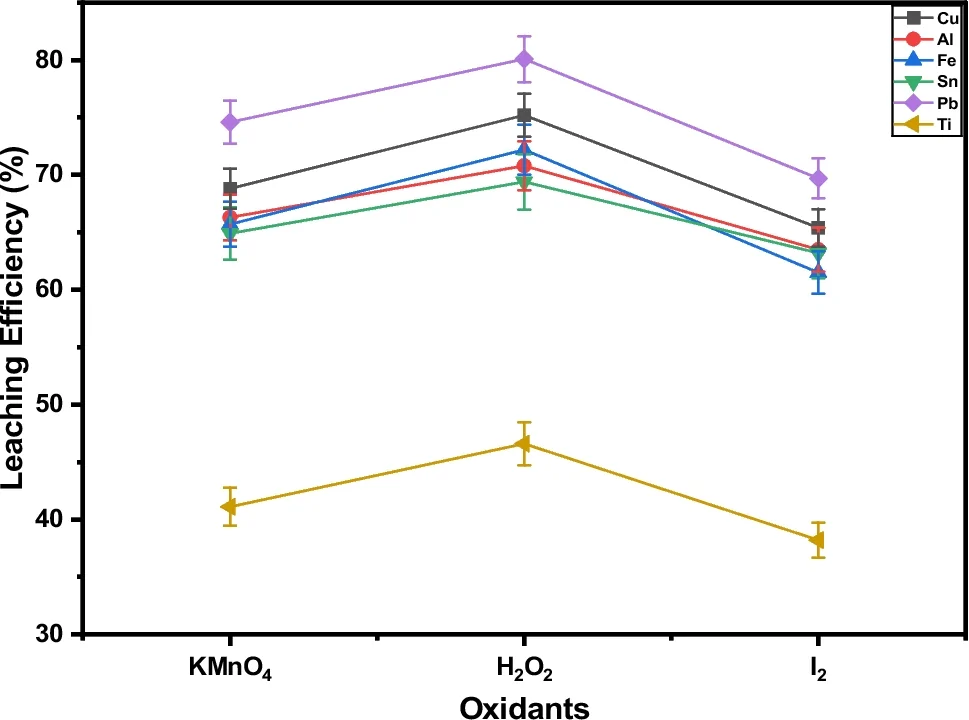
Effect of the type of oxidants on the leaching of metals using BET:ETD [T = 55 °C; t = 4 h; stirring speed = 500 & S:L = 1:20, oxidants = 1.0 M]

The incorporation of oxidising agents is essential for the effective leaching of metals from WPCB. These substances primarily serve to initiate the oxidation of metallic components on the board’s surface, thereby enabling their efficient transition into the lixiviant during the leaching process. The effect of oxidant type on the leaching efficiency of metals from WPCBs was investigated using BET:ETD NADES. The experiments were conducted under the same experimental conditions as the “Effect of HBD alkyl chain length in NADES” section (i.e., a temperature of 55 °C, a duration of 4 h, a stirring speed of 500 rpm, and an S:L ratio of 1:20). Three oxidants, KMnO₄, H₂O₂, and I₂, were examined at 1.0 M to assess their ability to assist metal dissolution. The results depicted in Fig. 6 and Table S2 reveal a clear dependency of metal leaching on the choice of oxidant. The system utilising H_2_O_2_ consistently achieved the highest leaching efficiencies across all targeted metals. Specifically, H_2_O_2_ yielded leaching efficiencies of 75.2% for Cu, 70.8% for Al, 72.2% for Fe, 69.4% for Sn, 80.1% for Pb, and 46.6% for Ti. In contrast, the use of I_2_ resulted in the lowest leaching efficiencies, with values of 65.4% (Cu), 63.5% (Al), 61.5% (Fe), 63.2% (Sn), 69.7% (Pb), and 38.2% (Ti). The KMnO_4_ system exhibited intermediate performance, with recoveries of 68.8% (Cu), 66.3% (Al), 65.7% (Fe), 64.9% (Sn), 74.6% (Pb), and 41.1% (Ti).

The superior performance of H_2_O_2_ may be attributed to its strong oxidative potential and its capacity to generate highly reactive oxygen species, such as hydroxyl radicals $(\cdot \mathrm{OH})$, which facilitate rapid oxidation of metallic states to their corresponding ionic forms (Jadhao et al. 2023). The presence of $\cdot \mathrm{OH}$ radicals^−^ significantly enhances the oxidative capacity of the medium, elevating the standard reduction potential to 2.38 V (Jadhao et al. 2021). This potent thermodynamic drive facilitates the rapid dissolution and efficient recovery of metallic components from the solid matrix. The decomposition of H_2_O_2_ is illustrated in Eqs. (2–4).

2 $$H_2O_{2(aq)} + e^- \rightarrow \cdot OH_{(aq)} + OH^-_{(aq)}$$

3 $$\cdot OH_{(aq)} + H^+_{(aq)} + e^- \rightarrow H_2O_{(l)}$$

4 $$2H_2O_{2(aq)}\rightarrow\:2H_2O_{(l)}+O_{2(g)}$$

Conversely, the lower efficiency of I_2_ could be ascribed to its weaker oxidative potential and selective oxidation kinetics, which limit the extent of metal dissolution (Oke and Potgieter 2025b). While KMnO₄ is a potent oxidant with a high reduction potential, its larger molecular structure and slower diffusion within the viscous DES medium restrict its interaction with metal surfaces, resulting in intermediate extraction efficiencies (Perea et al. 2021). In summary, the overall metal recovery efficiency as a function of oxidant type follows the descending order: H_2_O_2_ > KMnO4 > I_2_, underscoring the critical role of oxidant selection in optimising hydrometallurgical recovery processes from WPCB matrices.

#### Effect of time

**Fig. 7:**
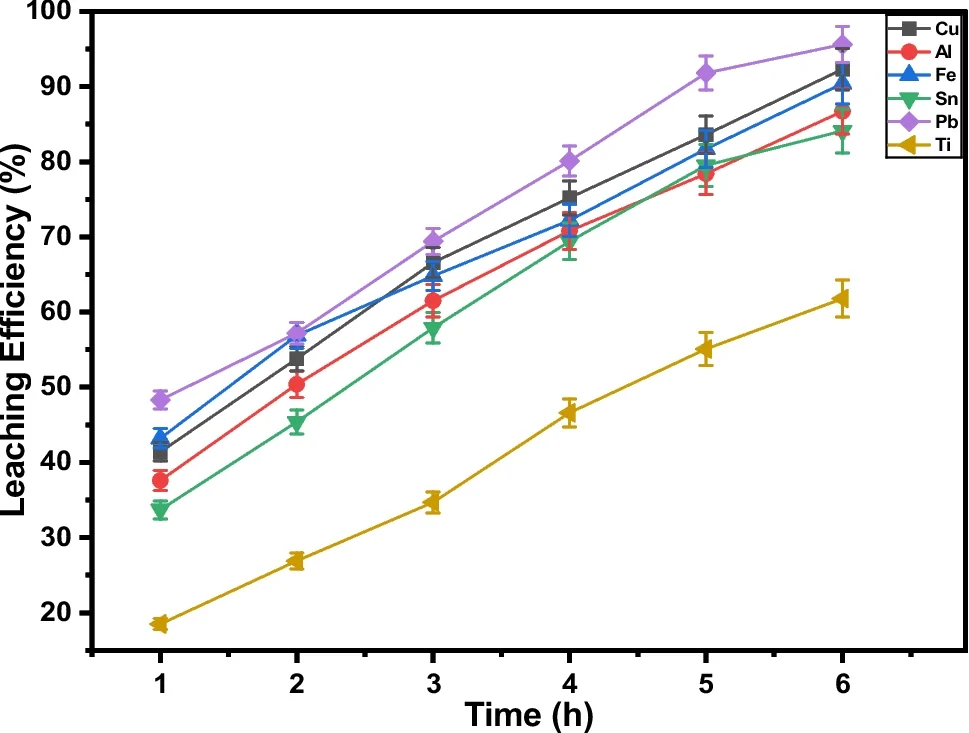
Effect of time on the leaching efficiency of metals using BET:ETD [T = 55 °C; t = 1–6 h; stirring speed = 500 & S:L = 1:20, H_2_O_2_ = 1.0 M]

Leaching time is a critical parameter governing the extent of metal extraction, as it directly influences the progression of metal dissolution and overall recovery efficiency. The effect of time was investigated from 1 to 6 h under fixed conditions of T = 55 °C, stirring speed = 500 rpm, S:L = 1:20, and H_2_O_2_ = 1.0 M using BET:ETD NADES. As the leaching time increased from 1 to 6 h, a consistent and substantial enhancement in extraction efficiency was observed for all metals (Cu, Al, Fe, Sn, Pb, and Ti) as demonstrated in Fig. 7 and Table S3. This is consistent with established hydrometallurgical principles where prolonged contact time enhances metal–solvent interactions and dissolution kinetics (Jadhao et al. 2023; Olaoluwa et al. 2023). For instance, Cu recovery increased from 41.4% at 1 h to 92.3% at 6 h, while Al improved from 37.6 to 86.7%. Fe and Sn followed similar trends, increasing from 43.2 to 90.4% and 33.7 to 84.1%, respectively. Pb exhibited the highest extraction efficiency across all time intervals, rising from 48.3 to 95.6%, whereas Ti showed the lowest recovery but still increased significantly from 18.5 to 61.8%.

During the initial stage (1–3 h), a sharp increase in extraction efficiency was observed for all metals. For example, Cu increased by ~ 25% (41.4 to 66.6%), Sn by ~ 24% (33.7 to 57.9%), and Pb by ~ 21% (48.3 to 69.4%). This rapid dissolution indicates that the process is strongly time-dependent at this stage, driven by a high concentration gradient, effective oxidant action, and the availability of abundant reactive surface sites. In the intermediate stage (3–5 h), the rate of increase remained significant but began to moderate. For example, Cu increased from 66.6 to 83.6% (~ 17% increment), while Pb rose from 69.4 to 91.8% (~ 22% increment). This behaviour suggests that although dissolution continues efficiently, the depletion of easily accessible metal phases and the onset of diffusion limitations begin to influence the kinetics (Shayakhmetova et al. 2025). Additionally, at longer durations (5–6 h), the increment in recovery becomes comparatively smaller for most metals. For instance, Cu increased by ~ 8.7%, Al by ~ 8.3%, and Fe by ~ 8.7%, indicating a transition toward a slower, diffusion-controlled regime. However, Ti continued to show a noticeable increase (55.1 to 61.8%), reflecting its relatively slower dissolution kinetics and stronger association within the solid matrix. Overall, leaching time exerts a pronounced influence on metal extraction, rapid initial dissolution followed by a gradual transition to slower kinetics. Since near-complete recovery (> 90.0%) is achieved for Cu, Fe, and Pb at 6 h; therefore, it was used for the subsequent experiments.

#### Effect of temperature

**Fig. 8:**
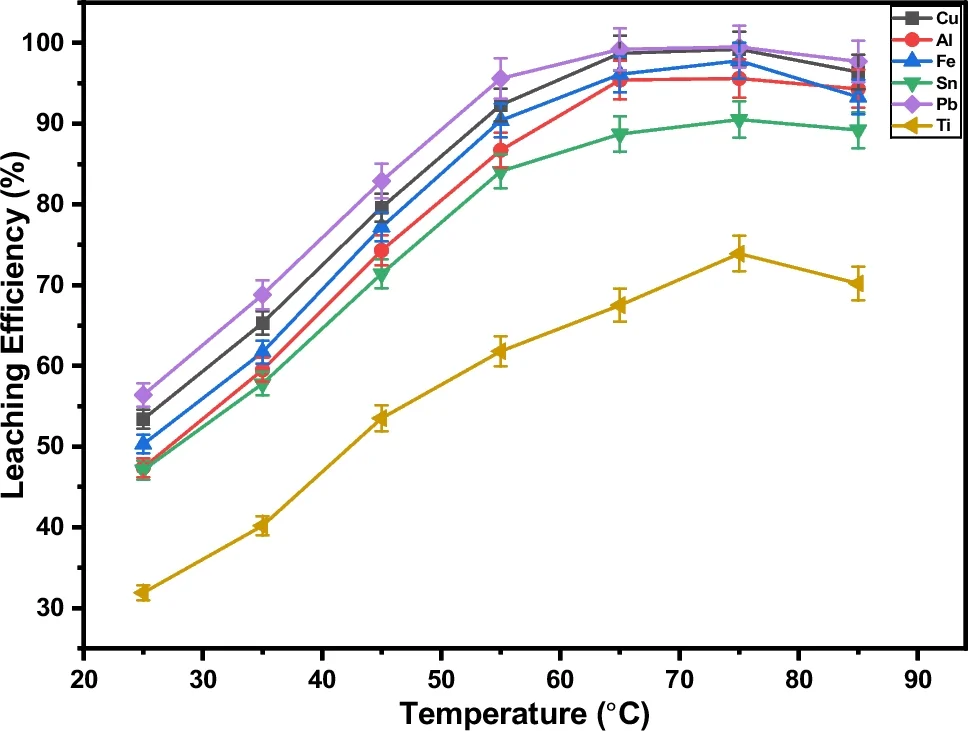
Effect of temperature on the leaching efficiency of metals using BET:ETD [*T* = 25–85 °C; *t* = 6 h; stirring speed = 500 & S:L = 1:20, H_2_O_2_ = 1.0 M]

Experiments were conducted by varying the temperature from 25 to 85 °C under fixed conditions of leaching time = 6 h, stirring speed = 500 rpm, S:L = 1:20, and H₂O₂ = 1.0 M using BET:ETD NADES. The results presented in Fig. 8 and Table S4 demonstrate that temperature plays a critical role in governing the leaching efficiency of Cu, Al, Fe, Sn, Pb, and Ti. As shown in Fig. 8 and Table S4, increasing the temperature from 25 to 65 °C resulted in a substantial enhancement in leaching efficiency for all metals. For example, Cu increased from 53.4 to 98.7%, Al from 47.4 to 95.4%, and Fe from 50.3 to 96.1%. Similarly, Sn and Pb increased from 47.1 to 88.7% and 56.2 to 99.2%, respectively, while Ti improved from 31.9 to 67.5%. This trend is attributed to improved reaction kinetics, reduced viscosity of the NADES, and enhanced mass transfer and diffusion of reactive species at elevated temperatures.

Between 65 and 75 °C, only marginal improvements were observed. For instance, Cu increased slightly from 98.7 to 99.3%, and Pb from 99.2 to 99.5%, indicating that the system is approaching near-complete dissolution and that further temperature increase provides limited kinetic benefit. At this stage, the process begins to shift toward diffusion limitations and depletion of readily accessible metal phases. Furthermore, at 85 °C, a noticeable decline in leaching efficiency was observed for all metals. For example, Cu decreased from 99.3 to 96.4%, Fe from 97.8 to 93.3%, and Pb from 99.5 to 97.7%. This reduction could be attributed to the accelerated decomposition of H_2_O_2_ at elevated temperatures, which reduces the availability of active oxidative species required for metal dissolution (Gubbins and Walker Jr 1965). Additionally, higher temperatures lower the solubility of dissolved oxygen in the system, further limiting oxidative pathways (Chen et al. 2015). The system may also approach a dynamic equilibrium at high temperature, where dissolution and re-precipitation of metal species occur simultaneously, leading to a net decrease in apparent leaching efficiency. Furthermore, rapid reaction rates at elevated temperatures can promote the formation of surface passivation layers, which hinder continued metal dissolution. Overall, temperature enhances leaching efficiency up to an optimal range (65–75 °C), beyond which oxidant instability and equilibrium effects dominate.

### Dissolution kinetics of metals

The rate of metal extraction is governed by the particle transport rate, the diffusion of both reactants and products through a porous product layer, and the reaction rate at the surface of the unreacted metal (Jadhao et al. 2021). In this study, it is assumed that the majority of metal ions are located within the particle core. The BET:ETD NADES facilitates metal leaching from the WPCB surface, which acts as the reaction zone. As the extraction period lengthens, this zone diminishes and progressively moves toward the particle interior. This process enables the leached metals to remain dissolved in the liquid phase alongside the residual solids. Accordingly, the shrinking core model (SCM) was adopted to identify the step governing the overall extraction rate from WPCB. Equation (5) presents the form of this model that assumes chemical reaction as the rate-limiting step (Mishra et al. 2024). Furthermore, the role of internal diffusion as the rate-controlling mechanism was examined. Equation (6) shows the SCM as applied to a diffusion-controlled reaction (Mishra et al. 2024).

5 $$1-{\left(1-x\right)}^\frac{1}{3}={\mathrm{k}}_{\mathrm{c}}t$$

6 $$1-3{\left(1-x\right)}^\frac{2}{3} +2(1-x)={\mathrm{k}}_{\mathrm{d}}t$$

In the above equations, k_c_ and k_d_ represent rate constants (h^−1^), t is reaction time (h), and x represents the reacted fraction of metals.

The applicability of the SCM to describe the dissolution kinetics of Cu, Al, Fe, Sn, Pb, and Ti in BET:ETD NADES was evaluated under the experimental conditions of temperature range 25–75 °C, leaching time of 0–6 h, stirring speed of 500 rpm, solid-to-liquid ratio of 1:20, and 1.0 M H_2_O_2_. The experimental data were fitted to both the chemical reaction-controlled model (Eq.5) and the internal diffusion-controlled model (Eq.6).

**Fig. 9:**
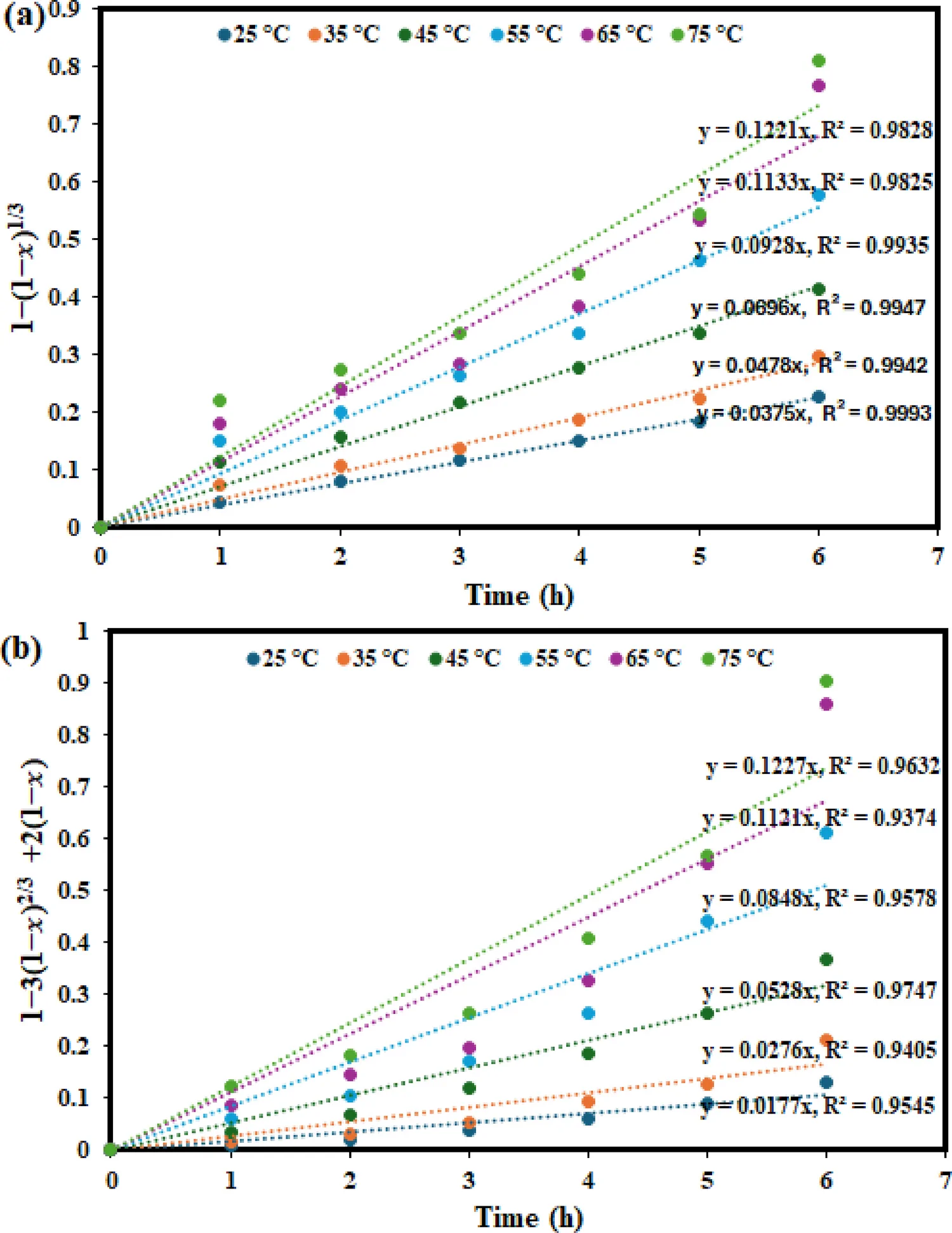
**a** and **b** Kinetic model plots based on the SCM for Cu leaching at different temperatures: **a** chemical reaction-controlled model and **b** diffusion-controlled model in BET:ETD NADES [Experimental conditions: 25–75 °C, 0–6 h, 500 rpm, S:L = 1:20, 1.0 M H_2_O_2_]

Representative kinetic plots for Cu are presented in Fig. 9a and b for the chemical reaction-controlled and diffusion-controlled models, respectively, while the corresponding fittings for all investigated metals are provided in the Supplementary Information (Fig. S1 and Fig. S2). For all metals, both models exhibited strong linear relationships with correlation coefficients (R^2^) approaching unity, indicating that either model can adequately describe the experimental data from a purely statistical standpoint. However, such comparable fitting quality suggests that linear regression alone is insufficient to uniquely identify the rate-controlling mechanism.

To resolve this ambiguity and further elucidate the rate-controlling mechanism, the apparent rate constants obtained from both kinetic models (Eqs.5 and 6) were used to determine the activation energy (E_a_) of the leaching process using the Arrhenius relationship (Eq.7), which provides key insight into the nature of the rate-limiting step governing the dissolution process.

7 $$\mathrm{lnk}= - \frac{\mathrm{E}\mathrm{a}}{\mathrm{R}} \left.\left( \frac{1}{\mathrm{T}}\right.\right) +\mathrm{l}\mathrm{n}\mathrm{A}$$

where k denotes the reaction rate constant, E_a_ represents the apparent activation energy (kJmol^−1^), R depicts the gas constant (8.314 J/mol/K), T symbolises the absolute temperature (K), and A is the pre-exponential factor.

**Fig. 10:**
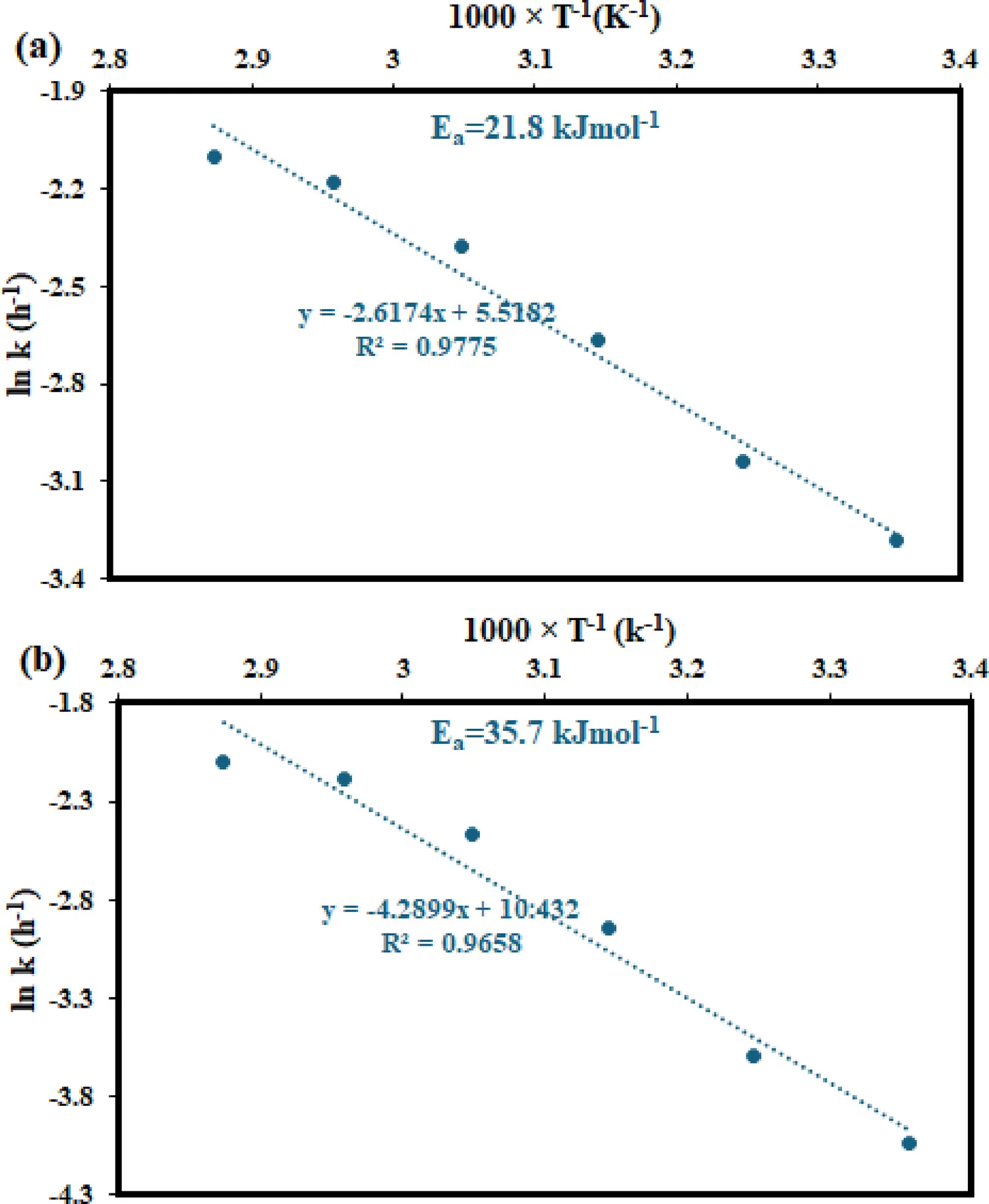
**a** and **b **Arrhenius plots for Cu extraction in BET:ETD NADES derived from **a** the chemical reaction-controlled model and **b** the diffusion-controlled model in BET:ETD NADES [Experimental conditions: 25–75 °C, 0–6 h, 500 rpm, S:L = 1:20, 1.0 M H_2_O_2_]

A representative Arrhenius plot for Cu is shown in Fig. 10a and b, while the corresponding plots for all metals are provided in the Supplementary Information (Fig. S3 and Fig. S4). The apparent activation energies (E_a_) calculated using the chemical reaction-controlled model were found to range from 21.3 to 23.5 kJ mol^−1^, whereas those derived from the diffusion-controlled model ranged between 35.7 and 38.8 kJ mol^−1^. From a mechanistic perspective, a surface chemical reaction-controlled process is typically associated with E_a_ values exceeding 40 kJ mol^−1^, while diffusion-controlled processes generally exhibit lower values, often between 4 and 12 kJ mol^−1^ (Han et al. 2020). In the present study, the E_a_ values obtained from both the chemical reaction and diffusion-controlled models fall outside the conventional ranges typically assigned to purely chemical or purely diffusion-limited processes.

Such deviations between SCM assumptions and experimentally derived activation energies have been widely reported in heterogeneous leaching systems, where multiple elementary steps may contribute simultaneously to the overall rate and single-mechanism descriptions become insufficient (Kumari et al. 2025). This inconsistency between model assumptions and E_a_ values therefore indicates that neither model independently governs the dissolution process. Instead, the extraction of Cu, Al, Fe, Sn, Pb, and Ti in BET:ETD NADES proceeds via a mixed-control mechanism, in which both interfacial chemical reactions and internal diffusion through the evolving product layer contribute concurrently to the overall rate. This behaviour is in agreement with the heterogeneous nature of WPCB, where metals are embedded within complex polymeric and metallic matrices, leading to spatially non-uniform accessibility of reactive sites. As leaching progresses, the formation of a porous product layer together with the gradual exposure of fresh reactive surfaces results in simultaneous contributions from surface reaction kinetics and mass transport limitations, as also inherent in the shrinking core framework. Furthermore, temperature increase within the investigated range enhances both diffusion coefficients and reaction rates through Arrhenius-type dependence, thereby preventing the dominance of a single rate-controlling step and sustaining a transition regime in which both mechanisms operate concurrently (Salinas-Rodriguez et al. 2022).

### Microstructure of WPCBs

**Fig. 11:**
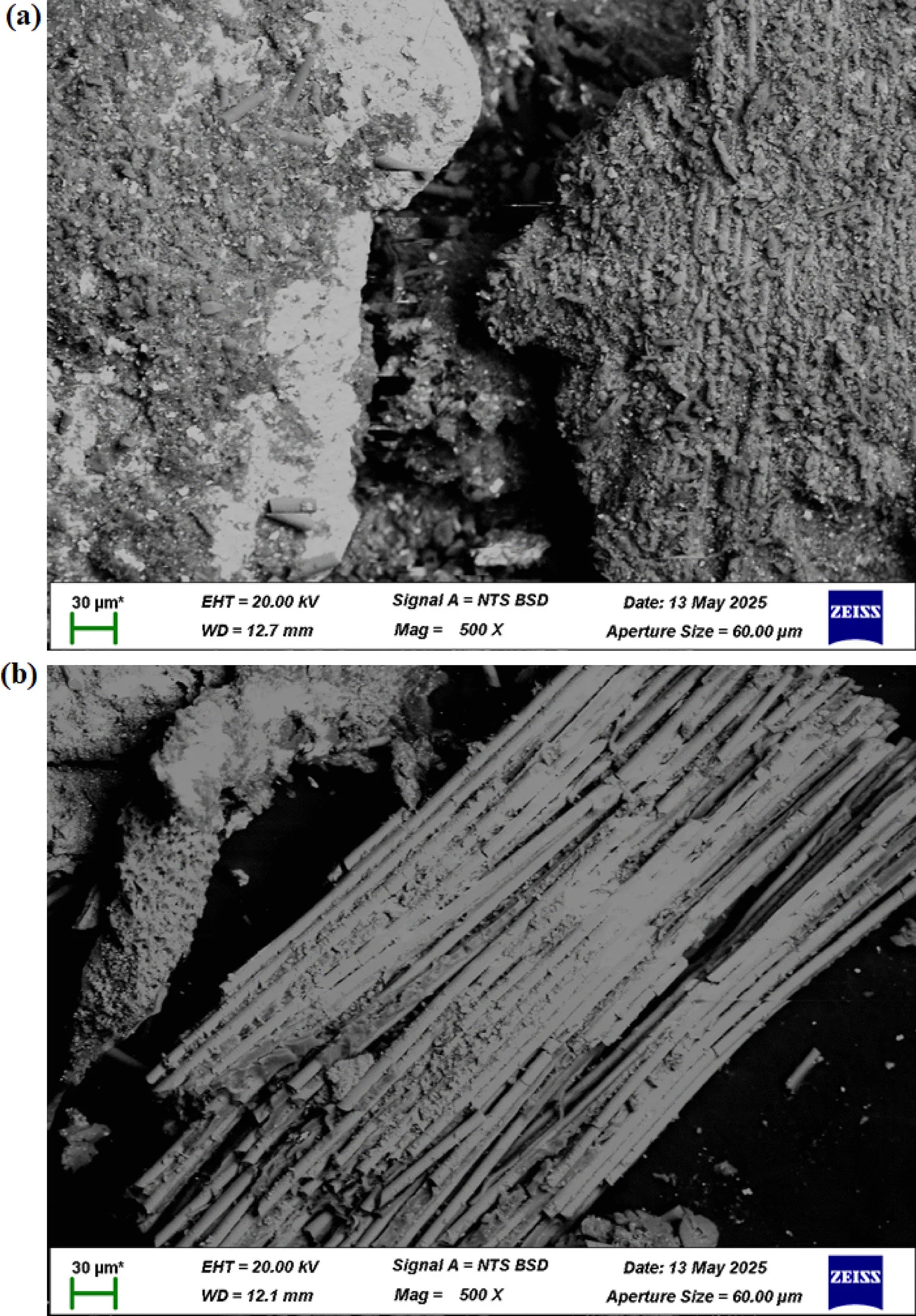
**a** and **b** SEM micrograph of **a** unleached WPCB and **b** the residue obtained after leaching

The morphological evolution of the WPCB samples before and after leaching was examined using SEM, with the results presented in Fig. 11a and b. The analysis captures the surface transformations induced by the BET:ETD NADES under experimental conditions of 75 °C, 6 h, 500 rpm, 1.0 M H_2_O_2_ and 1:20 S:L. The unleached WPCB (Fig. 11a) exhibits a highly heterogeneous and compact morphology, characterised by a dense arrangement of irregular flakes and fine spherical particles embedded within the leftover epoxy resin matrix. The surface appears relatively smooth and continuous, reflecting the encapsulation of metallic constituents such as Cu, Al, Fe, and Sn among others within brominated flame-retardant layers and the polymeric substrate.

After leaching, a pronounced structural transformation is observed in the residue (Fig. 11b). The combined action of the NADES and oxidant leads to substantial degradation of both organic and metallic phases, resulting in a markedly altered surface morphology. The previously compact structure is replaced by a more porous and irregular framework, indicating effective penetration of the solvent system into the WPCB matrix and subsequent dissolution of the targeted metal fractions. Moreover, a notable feature of the leached residue is the clear exposure of the underlying reinforcement structures, which appear as cylindrical rod-like formations corresponding to glass fibre bundles. These fibres, typically incorporated to provide mechanical strength in PCB fabrication, remain intact after leaching due to their silicate-based composition. Their visibility confirms the extensive removal of the surrounding resin and metallic components, leaving behind a skeletal framework. Similar observations were made by other authors in their studies (Annamalai and Gurumurthy 2021; Calgaro et al. 2015; Dutta et al. 2018). This morphological transition from a particle-dense, occluded surface to a clean, fibrous structure provides direct visual evidence of the effectiveness of the BET:ETD leaching platform. It also supports the proposed leaching mechanism, consistent with shrinking core or interfacial reaction models, where soluble phases are progressively dissolved while the insoluble matrix is retained.

### XRD and FTIR characterisation of the leaching residue

**Fig. 12:**
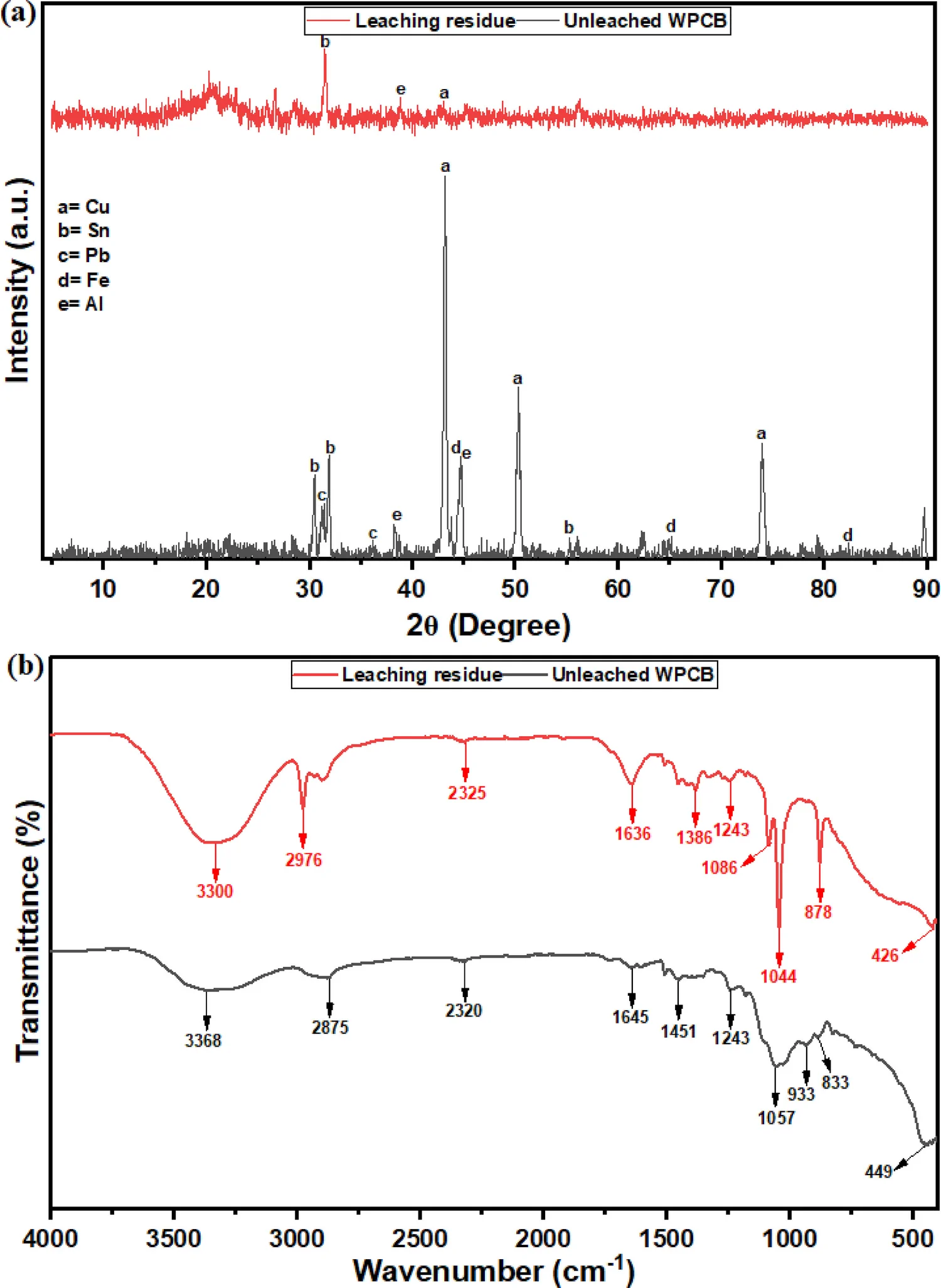
**a** XRD patterns and **b** FTIR spectra of the unleached WPCB and the residue obtained after leaching

The structural and chemical transformations associated with leaching were evaluated by comparing the unleached WPCB and the residue obtained after leaching using XRD and FTIR analyses, as illustrated in Fig. 12a and b. The characterisation of the leached residue was performed following metal extraction with the BET:ETD under experimental conditions of 75 °C, 6 h, 500 rpm, 1:20 S:L ratio, and 1.0 M H_2_O_2_.

The XRD analysis of the unleached WPCB and the residue obtained after treatment with the BET:ETD NADES lixiviant provides evidence of phase transformations and metal dissolution. Prior to leaching, the XRD pattern of the unleached WPCBs (Fig. 12) displays sharp, well-defined peaks at 2θ values corresponding to crystalline metallic phases of Cu, Sn, Pb, Fe, and Al. These peaks align with reference standards for Cu (PDF 01–085–1326) at 43.2°, 50.3°, and 74.0°, Al (PDF 01–089–2769) at 38.3° and 44.8° and Fe (PDF 03–065–4899) at 44.7°, 65.0°, and 82.3°, as established in our previous study (Oke and Potgieter 2025a). Furthermore, Sn exhibits prominent reflections at 30.5°, 31.9°, and 55.3° (PDF 03–065–0296), while Pb is identified by reflections at 31.3° and 36.3°. These findings are consistent with the dense morphology observed in SEM micrograph illustrated in Fig. 11, confirming the presence of a predominantly crystalline metallic structure before the leaching process. After leaching, the XRD pattern shows substantial reductions in peak intensities. The reflections corresponding to Cu, Fe, and Pb are no longer detectable, while Al peaks are significantly diminished, suggesting their near-complete dissolution into the lixiviant. This reduction in intensity suggests that several metallic peaks were amortised after the leaching process, indicating a clear disruption of the crystalline regions as metals were transferred from the alloy phase into the solution during digestion, a phenomenon similarly observed in studies using chemical and biological methods (Annamalai and Gurumurthy 2021; Kim et al. 2018; Zazycki et al. 2017). However, the Sn peaks remain relatively intense, particularly the reflection at 31.5°, indicating that Sn may not have completely leached under these conditions and remains as a residual crystalline phase. The overall reduction in peak intensity across the spectrum, coupled with the emergence of a broad amorphous hump between 15° and 25°, implies a significant decrease in crystalline order and the successful removal of the majority of the target metallic constituents.

Furthermore, the FTIR spectra presented in Fig. 12b illustrate the chemical transition from the unleached WPCB to the final leached residue, revealing the high stability of the organic and inorganic framework throughout the leaching process. The broad absorption bands at 3368 cm⁻^1^ in the unleached WPCB and 3300 cm⁻^1^ in the leaching residue are attributed to O–H stretching vibrations. According to Dutta et al., these bands signify the presence of hydroxyl groups in the substrate or residual moisture (Dutta et al. 2018). The slight shift and reduction in the broadness of this peak in the leaching residue suggest that the BET:ETD leaching process, involving the 1.0 M H_2_O_2_ oxidant, slightly modified the surface hydration or the hydrogen-bonding network of the exposed glass-reinforced matrix. Additionally, the peaks identified at 2875 cm⁻^1^ and 2976 cm⁻^1^ correspond to the C-H stretching vibrations of aliphatic groups, likely originating from the thermoplastic components like acrylonitrile butadiene styrene or polyethylene commonly found in e-waste (Arshadi et al. 2018).

Moreover, the mid-region signals at 1645 cm⁻^1^ (unleached) and 1636 cm⁻^1^ (leached) correspond to C=O stretching or N–H bending. As noted by Vakilchap et al., these peaks are often related to carboxylate groups or amides from the residual organic structures (Vakilchap et al. 2025). The persistence of these peaks in the leached residue demonstrates that the BET:ETD system is highly selective for metallic components and does not significantly react with the complex nitrogen-containing organic compounds or the remaining carbonaceous skeleton. Furthermore, the band at 1243 cm⁻^1^ found in both samples is associated with C-N stretching or C–O–C asymmetric vibrations. It was previously established by other authors that these bands represent the non-leachable plastic and ceramic fractions, reinforcing the conclusion that the polymer matrix remains structurally robust after metal extraction (Annamalai and Gurumurthy 2021; Dutta et al. 2018).

However, a closer look at the fingerprint region below 1000 cm⁻^1^ reveals the most significant evidence of the solvent’s selectivity. The intense absorption at 1057 cm⁻^1^ in the unleached WPCB and 1044 cm⁻^1^ in the leached WPCB is an indication of C-O stretching in esters or alcohols, which Annamalai and Gurumurthy linked to the fabrication materials of most WPCBs (Annamalai and Gurumurthy 2021). Consequently, the appearance of the sharp peak at 878 cm⁻^1^ in the leaching residue, compared to the weaker signal at 833 cm⁻^1^ in the unleached WPCB, suggests that the removal of metals and the action of the BET:ETD solvent further exposed the aromatic C-H bending of the secondary polymers. In contrast, the peaks observed at the lower frequency range, specifically between 426 cm⁻^1^ and 449 cm⁻^1^, are related to metal oxides or ceramic fillers. Vakilchap et al. observed that peaks below 700cm⁻^1^ often correspond to stable oxides (Vakilchap et al. 2025). Their presence in the leaching residue, as shown in Fig. 12b, confirms that while the target metals were leached, the more resilient inorganic oxides and the primary polymeric framework were preserved.

## Conclusions

This study demonstrates that the physicochemical properties of betaine-based NADES strongly govern their hydrometallurgical performance for metal recovery from WPCBs. Systematic variation of the HBD from ETD to BTD revealed clear differences in the physicochemical properties and leaching performance of the resulting NADES. Among the investigated NADES systems, BET:ETD exhibited the lowest viscosity at 20 °C (50.1 mPa·s), and it consistently displayed the highest leaching efficiencies among the three NADES under the comparative leaching conditions, indicating that the physicochemical characteristics of the NADES influence their leaching performance. Also, FTIR analysis confirmed the successful formation of extensive hydrogen-bonding interactions between BET and the diol-based HBDs, while TGA/DTG analysis showed that pronounced mass-loss-rate maxima occurred above approximately 240 °C for all three NADES. Rheological analysis further showed that the solvents exhibit stable Newtonian behaviour under the investigated conditions, supporting their suitability for hydrometallurgical applications requiring predictable flow and transport characteristics. Under the experimental conditions of 75 °C, 6 h, 1.0 M H_2_O_2_, 500 rpm, and a solid-to-liquid ratio of 1:20, BET:ETD achieved leaching efficiencies of 99.3% for Cu, 99.5% for Pb, 97.8% for Fe, and 95.4% for Al. Under the same conditions, Sn and Ti showed lower leaching efficiencies of 90.5% and 73.9%, respectively. The incorporation of H_2_O_2_ significantly enhanced metal dissolution due to its strong oxidative capability, establishing oxidant synergy as an essential factor in the overall extraction mechanism. Kinetic evaluation based on the shrinking core model and activation energy analysis revealed that the dissolution process proceeds through a mixed-control mechanism involving simultaneous interfacial reaction and diffusion phenomena. SEM, XRD, and FTIR analyses further confirmed substantial dissolution of metallic phases while preserving the polymeric and glass-fibre matrix of the WPCB substrate. Overall, this work provides important mechanistic insight into how HBD structure influences the physicochemical behaviour and extraction performance of BET-based NADES. The combination of high thermal stability, favourable rheological behaviour, strong extraction efficiency, and reduced dependence on aggressive mineral acids highlights the potential of these solvents as sustainable and tunable lixiviants for environmentally responsible e-waste recycling and urban mining applications.

## Supplementary Information

Below is the link to the electronic supplementary material.

ESM1 (DOCX 377 KB)

## Data Availability

The original contributions presented in the study are included in the manuscript. Further inquiries can be directed to the corresponding author.
