# Supplementary material for: Development of efficient betaine-based natural deep eutectic solvents for metal leaching from waste printed circuit boards
Source: Environ Sci Pollut Res Int. 2026 Oct 20;33(37):17535–54. doi: 10.1007/s11356-026-38292-9 (PMC13719871; doi:10.1007/s11356-026-38292-9)
Supplement: Supplementary file 1 [file 11356_2026_38292_MOESM1_ESM.docx]

**Development of Efficient Betaine-Based Natural Deep Eutectic Solvents for Metal Leaching from Waste Printed Circuit Boards**

Emmanuel Anuoluwapo Oke*, Johannes Hermanus Potgieter

Sustainable and Innovative Minerals and Metals Extraction Technology (SIMMET) Research Group, School of Chemical and Metallurgical Engineering, University of the Witwatersrand Johannesburg, Private Bag X3 PO Wits 2050, Johannesburg, South Africa.

*Corresponding Author: Emmanuel Anuoluwapo Oke

Email: emmanuel.oke@wits.ac.za or okeemmanuela@gmail.com

**Table S1.** Effect of HBD alkyl chain length in DESs on the leaching of metals [T= 55 °C; t= 4 h; stirring speed= 500 & S:L= 1:20].

| **NADES** | **Cu** | **Al** | **Fe** | **Sn** | **Pb** | **Ti** |
| --- | --- | --- | --- | --- | --- | --- |
| BET:ETD | 62.5±2.55 | 59.3±1.92 | 57.7±2.46 | 58.8±1.95 | 65.4±2.76 | 34.6±1.53 |
| BET:ETD | 60.1±1.84 | 54.9±1.74 | 54.4±2.34 | 51.6±2.10 | 61.2±2.44 | 33.5±2.08 |
| BET:BTD | 53.8±1.59 | 49.7±2.18 | 47.2±2.65 | 42.5±1.49 | 56.5±1.96 | 26.4±1.92 |

**Table S2.** Effect of the type of oxidants on the leaching of metals using BET:ETD [T= 55 °C; t= 4 h; stirring speed= 500 & S:L= 1:20, oxidants= 1.0 M].

| **Duration (h)** | **Cu** | **Al** | **Fe** | **Sn** | **Pb** | **Ti** |
| --- | --- | --- | --- | --- | --- | --- |
| KMnO_4_ | 68.8±2.52 | 66.3±1.91 | 65.7±2.17 | 64.9±1.88 | 74.6±2.87 | 41.1±2.14 |
| H_2_O_2_ | 75.2±2.28 | 70.8±2.12 | 72.2±2.42 | 69.4±2.19 | 80.1±2.00 | 46.6±1.86 |
| I_2_ | 65.4±1.99 | 63.5±2.05 | 61.5±1.95 | 63.2±2.21 | 69.7±2.25 | 38.2±1.75 |

**Table S3.** Effect of time on the leaching efficiency of metals using BET:ETD [T= 55 °C; t= 1−6 h; stirring speed= 500 & S:L= 1:20, H_2_O_2_= 1.0 M].

| **Time (h)** | **Cu** | **Al** | **Fe** | **Sn** | **Pb** | **Ti** |
| --- | --- | --- | --- | --- | --- | --- |
| 1.0 | 41.4±1.04 | 37.6±1.62 | 43.2±2.10 | 33.7±1.81 | 48.3±1.55 | 18.5±1.78 |
| 2.0 | 53.8±2.31 | 50.4±2.21 | 56.9±1.97 | 45.4±1.95 | 57.2±1.43 | 26.9±2.06 |
| 3.0 | 66.6±2.00 | 61.5±1.92 | 64.8±1.74 | 57.9±2.30 | 69.4±2.47 | 34.7±2.29 |
| 4.0 | 75.2±2.28 | 70.8±2.12 | 72.2±2.42 | 69.4±2.19 | 80.1±2.00 | 46.6±1.86 |
| 5.0 | 83.6±3.15 | 78.4±3.47 | 81.7±2.45 | 79.5±2.49 | 91.8±2.66 | 55.1±2.36 |
| 6.0 | 92.3±3.27 | 86.7±3.03 | 90.4±3.17 | 84.1±3.49 | 95.6±2.40 | 61.8±2.41 |

**Table S4.** Effect of temperature on the leaching efficiency of metals using BET:ETD [T= 25−85 °C; t= 6 h; stirring speed= 500 & S:L= 1:20, H_2_O_2_= 1.0 M].

| **Temperature (℃)** | **Cu** | **Al** | **Fe** | **Sn** | **Pb** | **Ti** |
| --- | --- | --- | --- | --- | --- | --- |
| 25 | 53.4±1.58 | 47.4±1.19 | 50.3±2.16 | 47.1±2.39 | 56.2±2.46 | 31.9±1.09 |
| 35 | 65.3±2.14 | 59.5±1.93 | 61.7±2.42 | 57.8±1.99 | 68.8±2.19 | 40.2±1.24 |
| 45 | 79.6±2.75 | 74.3±2.18 | 77.2±2.34 | 71.4±2.55 | 82.9±3.16 | 53.5±2.61 |
| 55 | 92.3±3.27 | 86.7±3.03 | 90.4±3.17 | 84.1±3.49 | 95.6±2.40 | 61.8±2.41 |
| 65 | 98.7±2.27 | 95.4±2.39 | 96.1±2.22 | 88.7±2.28 | 99.2±2.57 | 67.5±2.25 |
| 75 | 99.3±3.27 | 95.6±3.03 | 97.8±2.64 | 90.5±3.26 | 99.5±3.52 | 73.9±3.22 |
| 85 | 96.4±2.12 | 94.3±2.55 | 93.3±2.48 | 89.2±2.23 | 97.7±2.54 | 70.2±2.75 |

**Fig. S1**. SCM kinetic plots based on the chemical reaction-controlled model for the leaching of (a) Al, (b) Fe, (c) Sn, (d) Pb, and (e) Ti at different temperatures in BET:ETD NADES [Experimental conditions: 25–75 °C, 0–6 h, 500 rpm, S:L = 1:20, 1.0 M H_2_O_2_].

**Fig. S2**. SCM kinetic plots based on the diffusion reaction-controlled model for the leaching of (a) Al, (b) Fe, (c) Sn, (d) Pb, and (e) Ti at different temperatures in BET:ETD NADES [Experimental conditions: 25–75 °C, 0–6 h, 500 rpm, S:L = 1:20, 1.0 M H_2_O_2_].

**Fig. S3**. Arrhenius plots based on the chemical reaction-controlled model for the leaching of (a) Al, (b) Fe, (c) Sn, (d) Pb, and (e) Ti in BET:ETD NADES [Experimental conditions: 25–75 °C, 0–6 h, 500 rpm, S:L = 1:20, 1.0 M H_2_O_2_].

**Fig. S4**. Arrhenius plots reflect the diffusion-controlled model for the leaching of (a) Al, (b) Fe, (c) Sn, (d) Pb, and (e) Ti in BET:ETD NADES [Experimental conditions: 25–75 °C, 0–6 h, 500 rpm, S:L = 1:20, 1.0 M H_2_O_2_].
